# Propofol, an Anesthetic Agent, Inhibits HCN Channels through the Allosteric Modulation of the cAMP-Dependent Gating Mechanism

**DOI:** 10.3390/biom12040570

**Published:** 2022-04-12

**Authors:** Morihiro Shimizu, Xinya Mi, Futoshi Toyoda, Akiko Kojima, Wei-Guang Ding, Yutaka Fukushima, Mariko Omatsu-Kanbe, Hirotoshi Kitagawa, Hiroshi Matsuura

**Affiliations:** 1 Department of Anesthesiology, Shiga University of Medical Science, Otsu 520-2192, Japan; mshimizu@belle.shiga-med.ac.jp (M.S.); akiko77@belle.shiga-med.ac.jp (A.K.); yf@belle.shiga-med.ac.jp (Y.F.); hirotosi@belle.shiga-med.ac.jp (H.K.); 2 Department of Physiology, Shiga University of Medical Science, Otsu 520-2192, Japan; mi1990@belle.shiga-med.ac.jp (X.M.); toyoda@belle.shiga-med.ac.jp (F.T.); m_omatsu@belle.shiga-med.ac.jp (M.O.-K.); matuurah@belle.shiga-med.ac.jp (H.M.)

**Keywords:** hyperpolarization-activated cyclic-nucleotide gated channel, cyclic nucleotide binding domain, *I*_f_, propofol, cAMP

## Abstract

Propofol is a broadly used intravenous anesthetic agent that can cause cardiovascular effects, including bradycardia and asystole. A possible mechanism for these effects is slowing cardiac pacemaker activity due to inhibition of the hyperpolarization-activated, cyclic nucleotide-gated (HCN) channels. However, it remains unclear how propofol affects the allosteric nature of the voltage- and cAMP-dependent gating mechanism in HCN channels. To address this aim, we investigated the effect of propofol on HCN channels (HCN4 and HCN2) in heterologous expression systems using a whole-cell patch clamp technique. The extracellular application of propofol substantially suppressed the maximum current at clinical concentrations. This was accompanied by a hyperpolarizing shift in the voltage dependence of channel opening. These effects were significantly attenuated by intracellular loading of cAMP, even after considering the current modification by cAMP in opposite directions. The differential degree of propofol effects in the presence and absence of cAMP was rationalized by an allosteric gating model for HCN channels, where we assumed that propofol affects allosteric couplings between the pore, voltage-sensor, and cyclic nucleotide-binding domain (CNBD). The model predicted that propofol enhanced autoinhibition of pore opening by unliganded CNBD, which was relieved by the activation of CNBD by cAMP. Taken together, these findings reveal that propofol acts as an allosteric modulator of cAMP-dependent gating in HCN channels, which may help us to better understand the clinical action of this anesthetic drug.

## 1. Introduction

Propofol (2,6-diisopropylphenol) is an intravenous anesthetic drug that is widely used in the induction and maintenance of general anesthesia and sedation because of its advantages, which include a controllable state and fast awakening [1]. The potentiation of GABA_A_ receptor Cl^−^ channels has long been considered a major mechanism for the anesthetic action of propofol [2,3,4]. This drug also modulates the function of several voltage gated ion channels, including hyperpolarization-activated, cyclic nucleotide-gated (HCN) channels [5,6,7], which may also contribute to its principal anesthetic effects. It is also known that propofol infusion can cause several side effects (propofol infusion syndrome) in several organs, including myocardial cells, such as metabolic acidosis, hyperkalaemia, rhabdomyolysis, or signs of heart failure. A dosage reduction or discontinuation is recommended when these conditions developed [8]. On the other hand, propofol provides cardioprotection of cardiomyocyte function under ischemic condition e.g., in animal model of heart failure, propofol was reported to protect against peroxidative and functinal damages induce by exogenous H_2_O_2_ [9].

HCN channels control the electrical excitability and rhythmicity of neuronal and cardiac pacemaker cells by producing hyperpolarization-activated cationic inward current (*I*_h_ in neurons or *I*_f_ in the heart) [10]. In fact, mutation in hHCN4 is known to result in sinus bradycardia [11]. HCN channels allow Na^+^ influx in the cells under physiological situation, and an increase in channel activation might elevate intracellular Ca^2+^ transiently by a reverse mode of Na^+^/Ca^2+^ exchange current [12]. Therefore, in certain pathophysiological situations, the inhibition of HCN channels might protect against several damages from cardiac remodeling after myocardial infraction, such as cardiomyopathy and arrhythmia.

The blocking of *I*_h_ in central neurons may be associated with the general anesthetic action of propofol [5,13,14], whereas the *I*_f_ inhibition in cardiac pacemaker cells has been implicated in propofol-induced bradycardia [15]. Despite its fundamental importance, the molecular mechanism underlying the action of propofol on the HCN channels is poorly understood. The HCN channels (HCN1–4) are members of the voltage-gated cation channel superfamily that share a common six transmembrane-tetrameric architecture, consisting of one central pore domain (PD) surrounded by four peripheral voltage-sensor domains (VSDs). Unlike most other voltage-gated channels that are opened by membrane depolarization, the HCN channels are activated by membrane hyperpolarization. In addition, a distinguishing feature of HCN channels is the presence of cyclic nucleotide-binding domains (CNBDs) at their intracellular C-termini. Previous functional and structural analyses have suggested that CNBDs act as an inhibitory module of the inner pore gate [16,17,18,19,20]. Binding of cAMP to CNBDs can relieve the autoinhibition and thereby facilitate HCN channel opening. Thus, the opening of HCN channels is operated by both membrane voltage and cAMP, with distinct but allosterically coupled gating pathways [21,22]. Recent cryo-electron microscopy (EM) studies revealed molecular details of the structural elements of the PD, VSD, and CNBD, opening the door for the realization of the mechanics and dynamics of HCN channel gating [17,23]. This advanced knowledge on the structure and function provide an attractive framework for understanding the HCN channel behaviors as well as drug actions.

The present study investigated the effect of propofol on HCN channels in terms of their gating mechanism. For this purpose, we performed whole-cell patch clamp recordings to assess the drug responses of HCN4 and HCN2 channels in a heterologous expression system. In addition, to better interpret the drug effect, our experimental data were further analyzed using an allosteric gate model for HCN channels [22]. With these approaches, our results provide a better understanding of how propofol affects the gating properties of HCN channels.

## 2. Materials and Methods

### 2.1. Complementary DNA, Cell Culture, and Transfection

The full-length cDNA encoding human HCN4 (GenBank accession number NM_005477) and HCN2 (GenBank accession number NM_001194) channels, subcloned into the mammalian expression vector pcDNA3, ere a kind gift from Dr. Juliane Stieber (Lehrstuhl Pharmakologie und Toxikologie, Munich, Germany).

Chinese hamster ovary (CHO; RRID: CVCL_0213) cells were maintained in DMEM/Ham’s F-12 medium supplemented with 10% fetal bovine serum (Sigma Chemical Company, St Louis, MO, USA) and antibiotics (100 IU/mL penicillin and 100 µ/mL streptomycin) in a humidified atmosphere under 5% CO_2_ at 37 °C. The cells were passaged twice a week and a fraction of the cells was plated onto glass coverslips (3 × 5 mm^2^) in 35-mm culture dishes. Each HCN4 (0.5 µg) or HCN2 DNA (0.5 µg) was transiently transfected into CHO cells together with green fluorescent protein (GFP) cDNA (0.5 µg) using Lipofectamine (Invitrogen Life Technologies, Carlsbad, CA, USA). After transfection for approximately 48 h, the GFP-positive cells were used in the patch-clamp study.

### 2.2. Whole-Cell Patch-Clamp Recording and Data Analysis

Whole-cell membrane currents were recorded in voltage-clamp models with an EPC-8 patch-clamp amplifier (HEKA Elektronik, Lambrecht, Germany). Patch electrodes were fabricated from glass capillaries (outer diameter, 1.5 mm; inner diameter, 0.9 mm; Narishige Scientific Instrument Laboratory, Tokyo, Japan) using a Brown-Flamming microelectrode puller (model P-97, Sutter Instrument, Novato, CA, USA), and the tip was then fire-polished using an MF-830 microforge (Narishige, Tokyo, Japan). The electrodes had a resistance of 2.5–4.0 MΩ when filled with pipette solution. The liquid junction potential (approximately −10 mV) was compensated for. A glass coverslip with adherent CHO cells was placed in the chamber mounted on the stage of an inverted microscope (ECLIPSE TE2000-U, Nikon, Tokyo, Japan), and continuously perfused at a rate of 2 mL/min with normal Tyrode solution at 37 °C. Signals were low-pass filtered at 1 kHz, and acquired at 5 kHz through an LIH 1600 analogue-to-digital converter (Instrutech, NY, USA). The data were stored and analyzed using the Patchmaster software program (HEKA Elektronik).

The bath solution for whole cell recording was normal Tyrode solution containing (in mM) 140 NaCl, 5.4 KCl, 1.8 CaCl_2_, 0.5 MgCl_2_, 0.33 NaH_2_PO_4_, 5.5 glucose, and 5.0 HEPES (pH was adjusted to 7.4 with NaOH). The pipette solution contained (in mM) 70 potassium aspartate, 50 KCl, 10 KH_2_PO_4_, 1 MgSO_4_, 3 Na_2_-ATP (Sigma Chemical Company), 5 HEPES, 5 EGTA, 0.1 Li_2_-GTP (Sigma), and 2 CaCl_2_ (pH was adjusted to 7.2 with KOH). Propofol (Sigma) was dissolved in DMSO and then added to the bath solution at concentrations of 0.3, 1, 3, and 10 µM. Our previous studies have confirmed that DMSO as a solvent had no influence on the membrane ion currents in cell line and native cardiac cells when its concentration was between 0.04~0.1% (*v*/*v*) [24,25]. In the present study, the concentration of DMSO in the final solution was ≤0.002%. Cyclic adenosine 3′, 5′-monophosphate (cAMP, Sigma) was dissolved in pipette solution at a concentration of 50 µM.

The HCN2 and HCN4 currents were activated by 2-s hyperpolarizing voltage-clamp steps applied from a holding potential of −30 mV to test potentials of −40 to −150 mV in 10-mV increments, with each subsequent depolarizing step to 0 mV to record the current of deactivation. In each cell, the tail current amplitudes were measured and normalized to cell membrane capacitance to yield current density (*I*_tail_). The current density–voltage (*I*_tail_, density-*V*) relationships were fitted to a Boltzmann equation: *I*_tail_ = *I*_tail,max_/(1 + exp((*V*_t_ − *V*_h_)/*k*)), where *I*_tail,max_ is the maximal tail current density, *V*_h_ is the voltage at half-maximal activation, *V*_t_ is the test potential, and *k* is the slope factor. For the analysis of voltage dependence for the channel activation, the *I*_tail_, density-*V* relationships were all normalized to the maximum current density.

### 2.3. Model Simulation of Electrophysiological Data

The allosteric gate model for HCN channel activation [22] was used to analyze our patch clamp data. This model was developed by parameterizing the HA model [26] for the BK channel with the assumption that the allosteric coupling between the PD, VSD and CNBD was autoinhibitory. The two-state equilibrium behaviors of three functional modules of the PD, VSD, and CNBD were intrinsically characterized by distinct equilibrium constants, *L*, *H*(*V*)*,* and *K*, respectively. In the present study, these parameters were fixed as follows: (1) the PD close–open equilibrium constant, *L* = 85.3, in terms of an intrinsic bias towards to the open state, (2) the VSD resting-activated equilibrium constant, $H=H_{0}e^{\left(V_{t}/V_{h}\right)}$, where the zero voltage value $H_{0}=0.003$ and the half-activation voltage $V_{h}=-11.3\;\mathrm{mV}$, and (3) the CNBD cAMP binding constant $K=\left[\mathrm{cAMP}\right]/K_{D}$, where the dissociation constant $K_{D}=75\;µM$. The autoinhibitory coupling strengths of the PD with each of the four VSDs and four CNBDs were represented by allosteric factors *F* and *C*, respectively, such that the PD equilibrium constant decreased to a minimum of $L/(F^{4}C^{4})$ when both VSDs and CNBDs were resting. However, the PD equilibrium constant increased *F*-fold with the activation of each VSD and also *C*-fold with the cAMP binding to each CNBD to a maximum of *L*. Reciprocally, intrinsic equilibrium constants in the VSD and CNBD decreased to H/F and K/C, respectively, when the PD is closed. This autoinhibitory constraint was again removed by the opening of the PD. Similarly, the autoinhibitory mechanism was also adopted for functional interaction between the VSD and CNBD, with an allosteric factor *E.* This model consists of 70 states (Horrigan and Aldrich, 2002) and the steady state open probability ($P_{O}$) was calculated using the following equation:

(1) $$P_{O}=\frac{L/{\left(CF\right)}^{4}\times {\left(1+H/E+K/E+HK/E\right)}^{4}}{L/{\left(CF\right)}^{4}\times {\left(1+H/E+K/E+HK/E\right)}^{4}+{\left(1+H/\left(EF\right)+K/\left(CE\right)+HK/\left(CEF\right)\right)}^{4}}$$

The model was fitted to the experimental $P_{O}$-*V* data in the presence and absence (control) of propofol, in which values were normalized to the maximum available current density in control to yield the relative fraction of the open channel (i.e., relative $P_{O}$). The model fitting was performed using the Microsoft Excel Solver and parameter sets of allosteric factors, *F*, *C,* and *E,* were determined. The obtained parameters were employed in a simulation study. Given that stepwise activation of CNBDs occurs by the binding of cAMP to each of four subunits, the $P_{O}$ was simplified for individual states of CNBD activation as follows:

(2) $$P_{O}=\frac{L/{\left(CF\right)}^{4}\times {\left(1+H/E\right)}^{4}}{L/{\left(CF\right)}^{4}\times {\left(1+H/E\right)}^{4}+{\left(1+H/\left(EF\right)\right)}^{4}}$$

when none of the four CNBDs are activated.

(3) $$P_{O}=\frac{L/{\left(CF\right)}^{4}\times \left(1+H\right){\left(1+H/E\right)}^{3}}{L/{\left(CF\right)}^{4}\times \left(1+H\right)\times {\left(1+H/E\right)}^{3}+\left(1/C+H/\left(CF\right)\right)\times {\left(1+H/\left(EF\right)\right)}^{3}}$$

when one of four CNBDs is activated.

(4) $$P_{O}=\frac{L/{\left(CF\right)}^{4}\times {\left(1+H\right)}^{2}\times {\left(1+H/E\right)}^{2}}{L/{\left(CF\right)}^{4}\times {\left(1+H\right)}^{2}\times {\left(1+H/E\right)}^{2}+{\left(1/C+H/\left(CF\right)\right)}^{2}\times {\left(1+H/\left(EF\right)\right)}^{2}}$$

when two of four CNBDs are activated.

(5) $$P_{O}=\frac{L/{\left(CF\right)}^{4}\times {\left(1+H\right)}^{3}\times \left(1+H/E\right)}{L/{\left(CF\right)}^{4}\times {\left(1+H\right)}^{3}\times \left(1+H/E\right)+{\left(1/C+H/\left(CF\right)\right)}^{3}\times \left(1+H/\left(EF\right)\right)}$$

when three of four CNBDs are activated.

(6) $$P_{O}=\frac{L/{\left(CF\right)}^{4}\times {\left(1+H\right)}^{4}}{L/{\left(CF\right)}^{4}\times {\left(1+H\right)}^{4}+{\left(1/C+H/\left(CF\right)\right)}^{4}}$$

when all four CNBDs are activated.

### 2.4. Statistical Analyses

All data are expressed as the mean ± S.E.M. and the number of cells is indicated by n. Statistical comparisons were performed using the Student’s two-tailed paired or unpaired *t*-test or using ANOVA with the Tukey’s post hoc test (Prism Version 5.0), as appropriate. *p* values of <0.05 were considered to indicate statistical significance.

## 3. Results

### 3.1. Inhibitory Effects of Propofol on HCN4 Channels Expressed in CHO Cells

**Figure 1:**
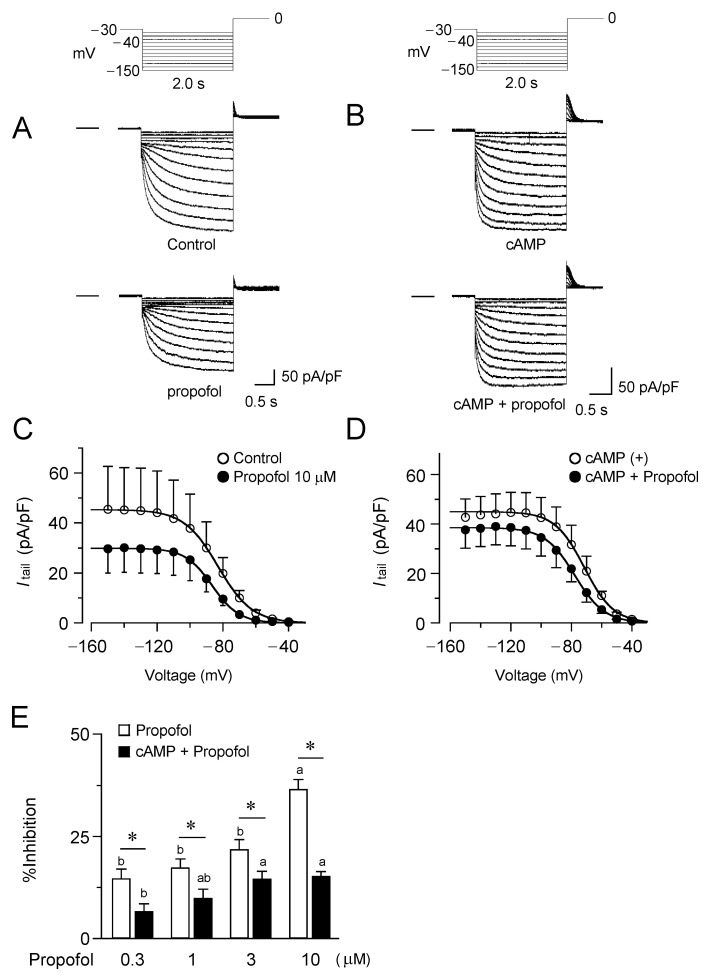
Inhibitory effects of propofol on HCN4 channels. (**A**) Superimposed current traces from HCN4-expressing CHO cells, recorded using the pulse protocol indicated above, before (upper, control) and 2–3 min after exposure to 10 μΜ propofol (lower). The horizontal line to the left of the current traces indicates zero level. (**B**) Current recordings in the cell preloaded with cAMP (50 μΜ) via pipette. The concentration of propofol is the same as A. (**C**) The mean *I*_tail_, density-*V* relationships for the current density in the presence and absence of propofol (*n* = 6). (**D**) The mean *I*_tail_, density-*V* relationships obtained in the presence of cAMP in the pipette (*n* = 6). (**E**) The percentage inhibition of the saturating tail current density at various concentrations of propofol in the presence and absence of cAMP. The data represent the mean ± S.E.M. (*n* = 6), and those followed by different letters indicate a significant difference at *p* < 0.05 according to the Tukey’s multiple range test and *t*-test (* indicates the difference in the presence and absence of cAMP; a, b, indicate different propofol concentration.).

The effect of propofol on the current property of HCN4 channels was examined using the whole-cell patch-clamp technique (Figure 1). Propofol was administered for 2–3 min, until it was ensured that the current had approached a steady state. As shown in a representative experiment in Figure 1A, propofol caused substantial inhibition of the HCN4 current; the effect was characterized by a reduction in the tail currents at 0 mV after hyperpolarizing voltage steps to different membrane potentials. The *I*_tail_, density-*V* relationships for tail currents revealed that the inhibition is at least due to a decrease in the saturating tail current density (Figure 1C). The inhibitory effect of propofol was dose-dependent (Figure 1E) and was even observed at clinically relevant plasma concentrations (~1.0 µM) [27]. The tissue concentration is estimated as 1.0 µM is approximately 27 µg/g in brain, 39 µg/g in liver, and 18 µg/g in kidney [27,28]. On average, 10 µM propofol produced a ~35% decrease in the current density determined after hyperpolarization to −120 mV.

**Figure 2:**
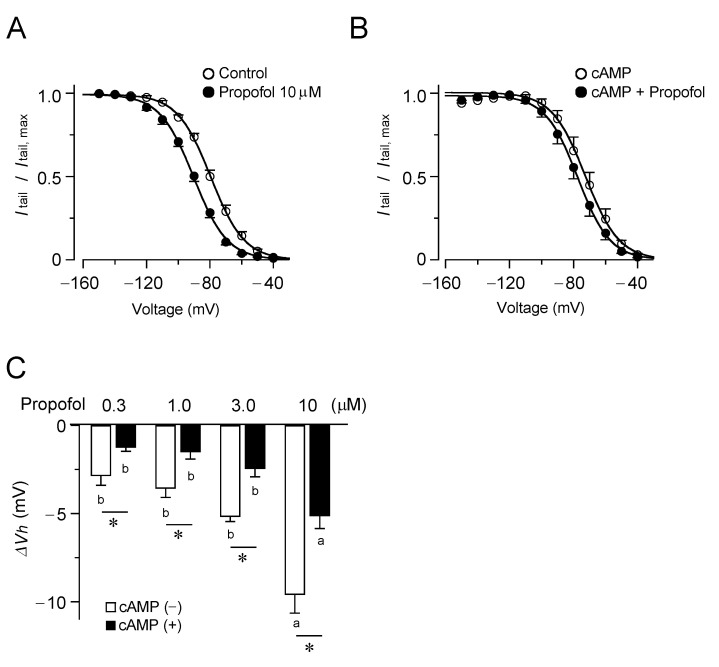
Propofol causes a hyperpolarizing shift in voltage dependence for HCN4 channel activation. The voltage dependence of HCN4 channel activation, in the presence and absence of propofol, was obtained from the cells using a pipette without (**A**) and with (**B**) cAMP. Tail current densities were normalized to the maximum value. The data points are mean ± S.E.M. (*n* = 6) and the smooth curves are the fit to the data using the Boltzmann equation. (**C**) Shifts in *V*_h_ caused by propofol at various concentrations in the presence and absence of cAMP. Bars represent the mean ± S.E.M. (*n* = 6), and those followed by different letters indicate a significant difference at *p* < 0.05 according to the Tukey’s multiple range test and *t*-test (* indicates the difference in the presence and absence of cAMP; a, b, indicate different propofol concentration.).

To examine the involvement of cAMP-dependent gating in the current inhibition by propofol, we recorded HCN4 currents in the presence of cAMP at a concentration of 50 µM in the pipette (Figure 1B). Measurements were taken >15 min after the start of cAMP loading to achieve the steady state. In agreement with previous reports on the HCN4 channels [29], cAMP caused a depolarizing shift in *V*_h_ (see also Figure 2A,B) with a slight but non-significant increase in the maximum current density (Figure 1D). As is evident in Figure 1B, in the presence of cAMP, propofol had sparing effect on the HCN4 currents. The inhibition of the tail current by propofol was significantly attenuated, and this attenuation was similar for all concentrations examined (Figure 1E), suggesting that cAMP influenced the propofol effect in a noncompetitive manner.

In Figure 2, the effect of propofol on the voltage dependence of HCN4 current activation was investigated. As illustrated in Figure 2A, propofol shifted the voltage-dependent opening of HCN4 channels to more hyperpolarized membrane potentials. This effect is important, as the hyperpolarizing shift in the activation threshold can profoundly reduce the current within the physiological range of membrane potentials [5]. The propofol-induced shift in the voltage dependence was evaluated with the changes in *V*_h_ (∆*V*_h_) at various concentrations, which expanded in a dose-dependent manner. We also examined these effects in the presence of cAMP (Figure 2B). Intracellular loadings of cAMP led to a shift in the voltage range of HCN4 channel activation toward more depolarized potentials (*V*_h_, −71 ± 0.99 mV and −82 ± 0.73 mV in the presence and absence of cAMP, respectively; *p* < 0.05). Subsequent application of propofol caused a slight shift in the activation curve to the opposite direction, but to a much lesser extent than that expected based on the observation without cAMP. In the presence of cAMP, propofol yielded significantly smaller ∆*V*_h_ values at all tested concentrations than that in the absence of cAMP (*p* < 0.05, Figure 2C).

### 3.2. Inhibitory Effects of Propofol on HCN2 Channels Expressed in CHO Cells

**Figure 3:**
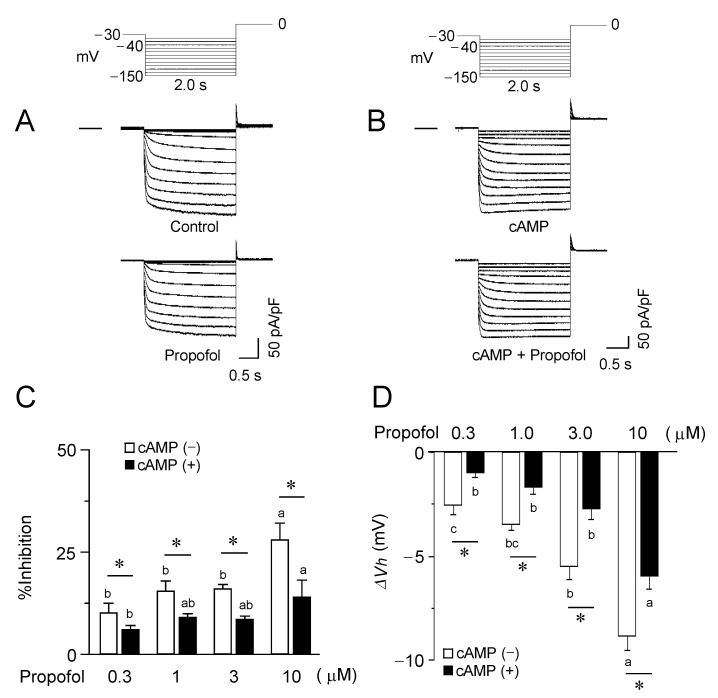
Inhibitory effects of propofol on HCN2 channels. (**A**) Superimposed current traces from HCN2-expressing CHO cells, recorded using the above-mentioned pulse protocol, before (upper, control) and 2–3 min after exposure to propofol at 10 μΜ (lower). The horizontal line to the left of the current traces indicates zero level. (**B**) Current recordings in a cell preloaded with cAMP (50 μΜ) via pipette. The concentration of propofol is the same as A. (**C**) The percentage inhibition of the saturating current density at various concentrations of propofol in the presence and absence of cAMP. Data represent the mean ± S.E.M. *, *p* < 0.05. (**D**) Shifts in *V*_h_ caused by propofol at various concentrations in the presence and absence of cAMP. Bars represent the mean ± S.E.M. (*n* = 6). The different letters in (**C**,**D**) indicate a significant difference at *p* < 0.05 according to the Tukey’s multiple range test and *t*-test (* indicates the difference in the presence and absence of cAMP; a, b, c, indicate different propofol concentration.).

It has been reported that propofol modulation varies among different isoforms of HCN channels [30]. Therefore, the same sets of experiments were carried out for the HCN2 channels (Figure 3). Consistent with previous reports [31], the HCN2 channels were activated more rapidly and at less negative potential in comparison to HCN4 channels. However, propofol exerted nearly identical effects on HCN2 channels in terms of the impact on the current density, as well as the voltage dependence for activation. Furthermore, in the presence of cAMP, propofol was less effective for modulating the functions of HCN2 channels, which was again similar to the observations for the HCN4 channels.

### 3.3. Computational Simulation of Propofol Effects on HCN4 Channels Using an Allosteric Gate Model

**Figure 4:**
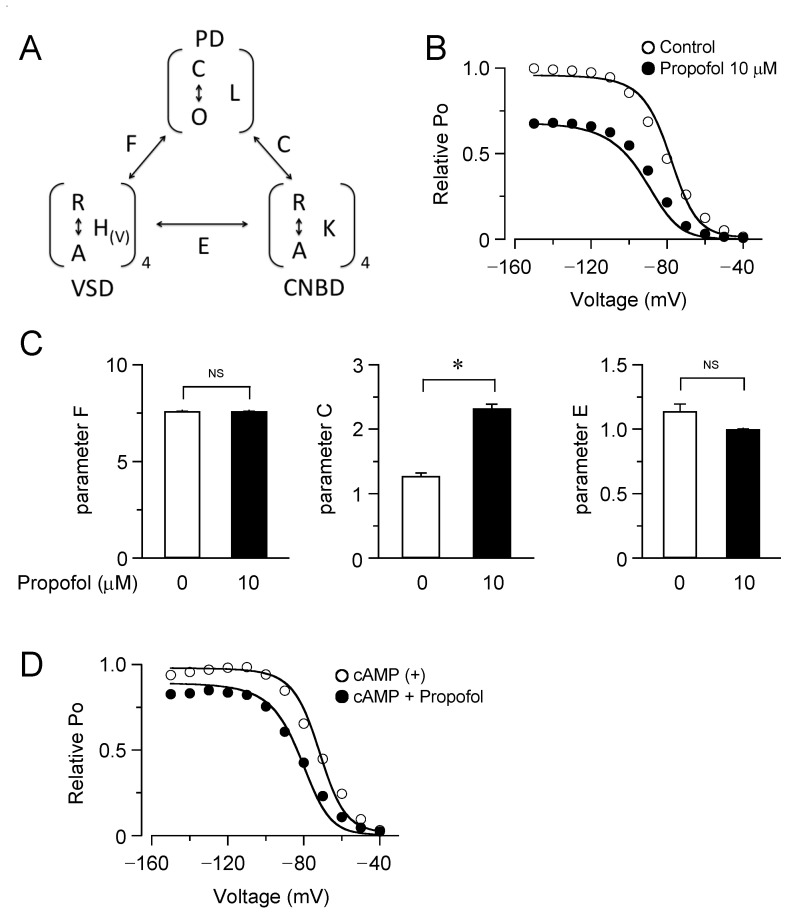
Allosteric gate model simulation of the inhibition of HCN4 channels by propofol. (**A**) The allosteric gate model for HCN channel activation, where *L*, *H(V)*, *K* are the equilibrium constants for the transitions between close and open (C–O) or rest and active (R–A) states in the PD, VSD, and CNBD, respectively. *F*, *C,* and *E* are the allosteric coupling factors between the PD and the VSD, between the PD and CNBD, and between the VSD and CNBD, respectively. (**B**) Model fitting to the $P_{O}$-*V* data in the presence and absence (control) of propofol, obtained by normalizing the current density (relative $P_{O}$ ) to the maximum value in control. The best-fit parameters of *F*, *C,* and *E* are 8.2, 1.6, and 1.6, respectively, in controls, and became 7.8, 2.9, and 1.3, respectively, in the presence of propofol. (**C**) Parameters of model fitting to the $P_{O}$ -*V* data in the presence and absence (control) of propofol. Bars represent the mean ± S.E.M. (*n* = 6). *, *p* < 0.05, NS, not significant. (**D**) Simulated $P_{O}$ -*V* curves with cAMP (50 μΜ) as determined by the model with the same parameter sets used in (**B**), which closely overlay the experimental data points.

**Figure 5:**
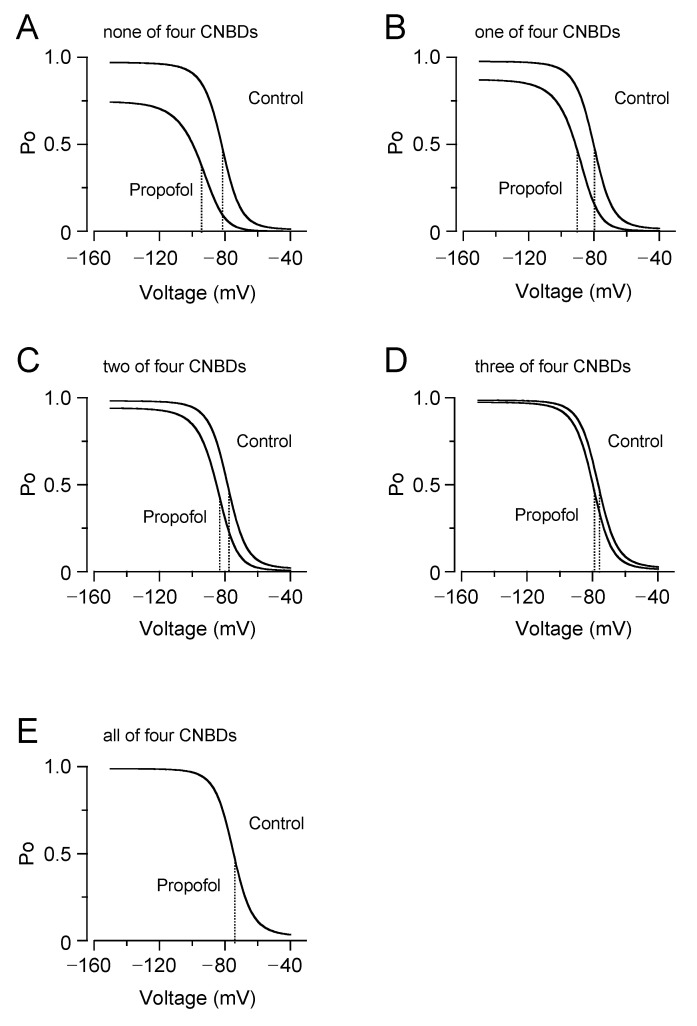
Simulation of the attenuated effects of propofol by activation of the CNBDs. The $P_{O}$-*V* curves in the presence and absence (control) of propofol were simulated depending on the activation state of CNBDs: none (**A**) and one to all (**B**–**E**) of four CNBDs activated. The vertical dashed line indicates the *V*_h_.

Our patch clamp data showed that the inhibition of the HCN channels by propofol was attenuated in the presence of cAMP, raising the question of whether and how cAMP-dependent gating is involved in the drug effect. To address this point, we employed an allosteric gating model for HCN channels, which was developed by Flynn and Zagotta [22]. This model includes autoinhibitory interactions between the PD, VSDs, and CNBDs, and the coupling strengths are represented by distinct parameters of *F*, *C,* and *E* (Figure 4A). The model fits the experimentally obtained plots of the relative tail current amplitudes ($P_{O}$-*V* curves) for HCN4 channels in the presence and absence of propofol (Figure 4B). The best-fit parameters revealed that propofol significantly increased the coupling factor between the PD and CNBDs, without changing other coupling strengths (Figure 4C). Results were validated by evaluating the sensitivity and specificity for each parameter (Figure S1, Supplementary Materials). Using the obtained parameter sets, the $P_{O}$-*V* curves were simulated with cAMP at 50 µM, where the equilibrium constant for the CNBD activation (*K* was assumed to be 0.667). As shown in Figure 4D, cAMP caused a depolarizing shift in the $P_{O}$-*V* curve. Furthermore, the model successfully reproduced the lesser effect of propofol on the $P_{O}$-*V* curve in the presence of cAMP. To gain further insight into these results, the $P_{O}$-*V* curves were generated by a simplified model, in which the activation of CNBD was fixed in a given state (Figure 5). The simulation clearly illustrated that the effects of propofol—both of the reduced maximum $P_{O}$ and the hyperpolarizing shift in the $P_{O}$-*V* curve—were gradually attenuated by the stepwise activation of four CNBDs and eventually abolished when the CNBDs were fully activated.

## 4. Discussion

Several anesthetic agents inhibit the HCN channel function [15,16,32,33,34]. However, their mechanism of action remains largely elusive, which is at least due to the complex nature of HCN channel gating. In our patch clamp study, inhibition of the HCN channels by propofol was characterized by a decrease in the maximum current density and a hyperpolarizing shift of the voltage dependence of channel opening, in accordance with previous studies [30,35]. Most importantly, we found that these effects were considerably attenuated in the presence of cAMP, suggesting the involvement of cAMP-dependent gating in the action of propofol. Indeed, the model fitting analysis showed that propofol primarily affected allosteric coupling between the PD and CNBDs to exert its inhibitory effect, providing a key to understanding the complicated effects of propofol on the HCN channels. The relief of autoinhibitory interactions between the PD and CNBDs has been suggested to be a mechanism for cAMP-dependent gating in the HCN channels. In contrast to CNG channels that typically require the cyclic nucleotides for their opening [36], HCN channels are principally opened by the voltage, even without cAMP. Thus, the autoinhibition is weak and incomplete in the HCN channels. In the present study, we found that propofol increased the coupling strength between the PD and CNBDs, indicating the enhanced tonic inhibition of the pore opening by unliganded CNBDs. This would account for the reduction of the maximum current by propofol. In addition, the enhanced autoinhibition can allosterically reduce the open fraction via the voltage-dependent gating, resulting in a hyperpolarizing shift in the activation curve. Considering that the autoinhibition is relieved by the activation of the CNBD, the effect of propofol could be attenuated in a graded manner with increased cAMP levels, while the increased coupling strength would greatly accentuate the cAMP-dependent facilitation of the HCN channels. Collectively, our findings illustrate how propofol acts as an allosteric modulator of cAMP-dependent gating in the HCN channels.

The site of action for propofol is still unidentified at a structural level. However, given its effects on cAMP-dependent gating, we hypothesize that the relevant structure resides between the PD and CNBDs, such as the C-linker. Previous functional and structural studies have revealed that the C-linker is critical for transmitting cAMP-induced conformational changes in the CNBD to the PD. Recently, a novel ligand-binding pocket was identified at the boundary between the C-linker and the CNBD [37]. The occupancy of this pocket by exogenously applied compounds such as cyclic dinucleotides [38] and TRIP8b_nano_ [39] interferes with the transmission of movement from the CNBD to the C-linker, thereby suppressing the responsiveness of HCN channels to cAMP. These findings provide the example of the allosteric regulation of the cAMP-dependent gate via the C-linker. Of note, the C-linker region is involved in the inhibitory effects of various drugs, including anesthetics [40]. Chen et al. [40] reported that HCN isoform-specific sensitivity to inhibition by halothane is imposed by differences in their structural arrangements of the C-linker domains. It is worth mentioning that the effect of halothane can be relieved by cAMP, similar to that observed for propofol in the present study. Besides, there is a growing body of structural evidence for a direct interaction of the CNBD with VSD though the HCN domain [41]. In this regard, we consider that the coupling factor *E* in the model may reflect a functional role of the HCN domain. Although disabled in the earlier study by Flynn and Zagotta [22], this parameter was included as a variable in the present study. However, in our model analysis, *E* was nearly one and was hardly affected by propofol. Further studies may illustrate the significance of functional coupling between CNBD and VSD in the HCN channels.

Given the fundamental role of the HCN channels in cardiac pacemaking, the inhibition of the HCN channel activity has been implicated as a cause of propofol-associated bradycardia [16]. According to the ACC/AHA/HRS guidelines [42], parasympathetic blockade with intravenous atropine is recommended for reducing bradycardia during the induction of total intravenous anesthesia with propofol. In light of recent experimental evidence from genetic mouse studies that investigated the HCN channels, this treatment seems to be reasonable. HCN4 knockout mice showed recurrent sinus pause and bradycardia [43,44,45], which typically manifested following vagal stimulation [46]. Interestingly, this pacemaker dysfunction was almost totally rescued by genetic ablation of the G-protein-coupled inward rectifier K^+^ channel, a primary contributor to the negative chronotropic response to muscarinic acetylcholine receptor stimulation [47]. Meanwhile, in the clinical setting, β-adrenergic agonists, such as isoproterenol, are also administered for severe bradycardia after propofol induction. This would also make sense because cAMP may exceedingly facilitate HCN channel opening by the activation of CNBDs, relieving the inhibition by propofol, as suggested by our observations. Accordingly, a clinical study reported that propofol anesthesia enhanced the heart rate increase in response to isoproterenol infusion [48]. In addition, cAMP may also cause cAMP-induced arrhythmia when it is accumulated [49,50]. Caution should however be exercised in interpreting such results in terms of the altered ionic mechanism underlying pacemaker activity, as well as the diverse pharmacological activity of propofol. β-adrenergic stimulation potentiates not only HCN-mediated *I*_f_ but also other ionic currents, including Ca_V_1.3-mediated *I*_Ca,L_ [51] and *I*_st_ [52,53,54], and K_V_7.1-mediated *I*_Ks_ [25], which are also potential targets of propofol [16]. Moreover, propofol significantly reduces sympathetic nerve activity [55].

## 5. Conclusions

Our patch clamp recordings and model fitting analyses of allosteric gating revealed that the anesthetic agent propofol inhibits HCN channels by functionally interacting with the cAMP-dependent gate. The model predicted that propofol facilitated the autoinhibition of pore opening by unliganded CNBDs, which could be relieved by cAMP. The model-based approaches provide a perspective for examinations and understanding the functional interactions of HCN channels with allosteric modulators.

## Data Availability

The data that support the findings of this study are available from the corresponding author upon reasonable request.

## Supplementary Materials

The following supporting information can be downloaded at: https://www.mdpi.com/article/10.3390/biom12040570/s1, Figure S1: Effects of changing each parameter on the $P_{O}$-*V* relationship.
