# Supplementary material for: Propofol, an Anesthetic Agent, Inhibits HCN Channels through the Allosteric Modulation of the cAMP-Dependent Gating Mechanism
Source: Biomolecules. 2022 Apr 12;12(4):570. doi: 10.3390/biom12040570 (PMC9032835; doi:10.3390/biom12040570)
Supplement: Supplementary file 1 [file biomolecules-12-00570-s001.zip › biomolecules-1672706-supplementary.pdf]

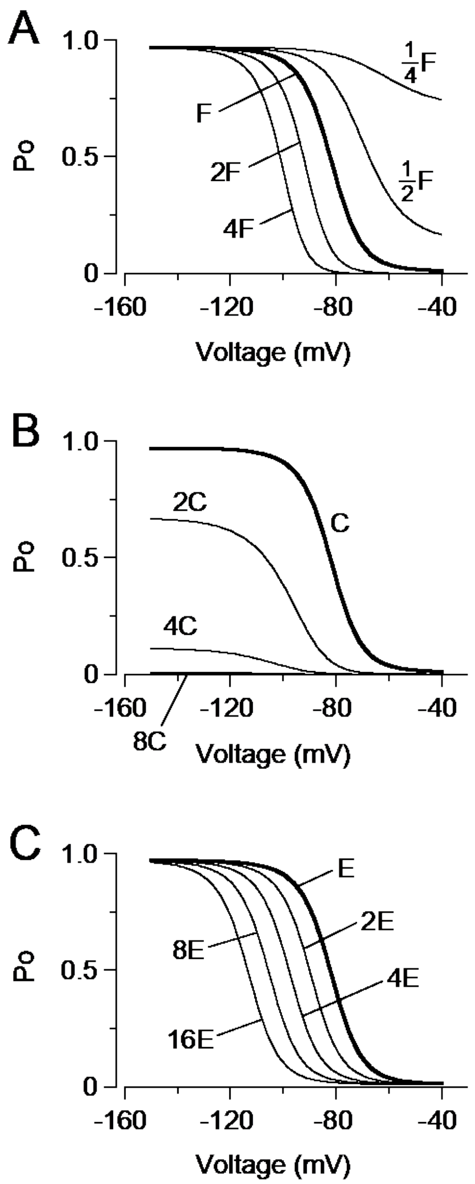

Figure S1. Effects of changing each parameter on the P_O- V relationship

The sensitivity and specificity of individual parameters were examined by changing an allosteric coupling factor *F* (A), *C* (B), or *E* (C) with the other two factors being fixed. The P_O- V curve obtained for the control experiments was used as a reference (thick line)
